# An Observational Study of Therapeutic Hypothermia and Factors Associated With Mortality in Late-Preterm and Term Neonates With Hypoxic-Ischemic Encephalopathy in a Middle-Income Country

**DOI:** 10.3389/fped.2022.894735

**Published:** 2022-06-10

**Authors:** Nem Yun Boo, Siew Hong Neoh, Seok Chiong Chee, for the Malaysian National Neonatal Registry

**Affiliations:** ^1^Department of Population Medicine, Faculty of Medicine and Health Sciences, Universiti Tunku Abdul Rahman, Bandar Sungai Long, Malaysia; ^2^Department of Paediatrics, Hospital Tunku Azizah, Ministry of Health, Kuala Lumpur, Malaysia; ^3^Department of Paediatrics, Selayang Hospital, Ministry of Health, Batu Caves, Malaysia

**Keywords:** hypoxic-ischemic encephalopathy (HIE), therapeutic hypothermia (TH), late-preterm and term neonates, middle-income countries, risk factors of mortality

## Abstract

**Objectives:**

To investigate the types of therapeutic hypothermia (TH) used and risk factors associated with mortality in late-preterm and term neonates (LPTN, gestation of ≥35 weeks) with hypoxic-ischemic encephalopathy (HIE) in a middle-income country.

**Design:**

This was an observational retrospective cohort study.

**Setting:**

A total of 44 neonatal intensive care units (NICUs) in the Malaysian National Neonatal Registry participated in the study.

**Patients:**

All LPTN without major malformations and diagnosed to have HIE were included.

**Main Outcome Measures:**

Number of in-hospital mortality, and types of TH used [no TH, TH using commercially available servo-controlled devices (SCDs), passive TH by exposing neonates to NICU’s air-conditioned ambient temperature with/without the use of cooled gel packs (P±CGPs)].

**Results:**

Of a total of 2,761 HIE neonates, 66.3% received TH. All NICUs provided TH; 55.4% NICUs had SCDs, which was administered to 43.6% (248/569) of severe, 51.6% (636/1,232) of moderate, and 18.6% (179/960) of mild HIE neonates. P±CGPs was used on 26.9% of severe, 33.4% of moderate, and 21.1% of mild HIE neonates. There were 338 deaths. Multiple logistic regression analysis showed that 5-min Apgar scores <5 (aOR: 1.436; 95% CI: 1.019, 2.023), Cesarean section (aOR: 2.335; 95% CI: 1.700, 3.207), receiving no TH (aOR: 4.749; 95% CI: 3.201, 7.045), TH using P±CGPs (aOR: 1.553; 95% CI: 1.031, 2.338), NICUs admitted <50 HIE cases (aOR: 1.898; 95% CI: 1.225, 2.940), NICUs admitted 50-<100 HIE cases (aOR: 1.552; 95% CI: 1.065, 2.260), moderate HIE (aOR: 2.823; 95% CI: 1.495, 5.333), severe HIE (aOR: 34.925, 95% CI: 18.478, 66.012), Thompson scores of 7–13 (aOR: 1.776; 95% CI: 1.023,3.082), Thompson scores of ≥14 (aOR: 3.641; 95% CI: 2.000, 6.629), pneumothorax (aOR: 3.435; 95% CI: 1.996, 5.914), and foreigners (aOR: 1.646; 95% CI: 1.006, 2.692) were significant risk factors associated with mortality.

**Conclusion:**

Both SCD and P±CGP were used for TH. Moderate/severe HIE and receiving passive/no TH were among the risk factors associated with mortality.

## Introduction

Neonatal hypoxic-ischemic encephalopathy (HIE) is due to acute peripartum or intrapartum events causing cerebral ischemia and oxygen deprivation. In the subsequent 1–6 h, oxidative metabolism may be restored with partial recovery of cells or secondary energy failure may continue leading to an increase in cerebral inflammation and cell death. Untreated, cytotoxic edema, excitotoxicity, and secondary energy failure develop with further cell death, seizures, clinical deterioration, and death (1–3). Survivors with moderate and severe HIE often develop long-term neurological impairments (4).

Therapeutic hypothermia (TH) at a moderate core temperature of 33.5 ± 0.5°C, initiated within 6 h after birth and maintained for 72 h, has been shown to reduce cellular injuries and cell death. Meta-analysis of studies in high-income countries (HICs) reported improved survival, following TH in late-preterm and term neonates (LPTN, gestation of ≥35 weeks) with moderate and severe HIE (5–8). The standard equipment used is a commercially available servo-controlled device (SCD) consisting of either a cooling blanket or cap with a thermostatic mechanism that rapidly cools the neonate to a target rectal temperature of 33.5 ± 0.5°C and maintains it continuously for 72 h (9). At the end of 72 h, the neonate is rewarmed gradually to normothermia over 12 h, as too rapid rewarming can result in worsening of HIE and seizures (10). However, not all NICUs in low- and middle-income countries (LMICs) are equipped with SCD as it is very costly (19750–20000 Euros per set). In recent years, some NICUs in LMICs reported improved survival of moderate and severe HIE neonates following TH using low-cost cooled gel packs (11).

In Malaysia, HIE is common (12). Many Malaysian NICUs started to administer TH on HIE neonates in 2010. To ensure the appropriate use of TH, the Malaysian Ministry of Health (MOH) published a practice guideline (13), which recommended TH to be commenced within 6 h after birth for 72 h on stabilized neonates with moderate or severe HIE and Thompson score (14) ≥7 or seizures. Other criteria which must be met are the presence of at least two of the following: Apgar score <6 at 10 min or needing prolonged resuscitation, presence of acute perinatal events, cord pH of <7 or base deficit of ≥12 mmol/L, or arterial pH < 7.0 or base deficit >12 mmol/L within 60 min of birth. The methods/devices approved for TH were a servo-controlled cooling blanket (SCD-blanket), a servo-controlled cooling cap (SCD-cap), or passive cooling with/without the use of cool gel packs (P±CGPs). SCD was the preferred method, and P±CGP was to be used when SCD was not available. Passive cooling was described as exposing a neonate (without clothing and cap) in a cot to the NICU’s air-conditioned ambient temperature (average 22–24°C). If the rectal temperature of the neonate dropped below 33.5°C, the radiant warmer would be used to maintain the rectal temperature at 33–34°C or axillary temperature at 33.5–34.5°C. If the neonate’s temperature remained >35°C despite 60 min of passive cooling, cooled gel packs (CGPs) would be used to provide additional cooling. Before use, gel packs (about 4 Euro per pack, bought from local pharmacies) were first cooled with refrigeration without being frozen. They were placed in cotton bags on the neonate under the shoulders or upper back or across the chest.

The Malaysian National Neonatal Registry (MNNR) was established in 2005 and by 2019 had 44 members (comprising all major MOH hospitals, one university hospital, and three private hospitals). Participation in the MNNR was voluntary. Member NICUs prospectively submitted data of neonates using a standardized data form to the MNNR. Since 2012, all LPTNs admitted to these centers and diagnosed to have HIE have been included in the database. Since 2016, information on types of TH administered to each HIE LPTN was also included. This study aimed to investigate the types of therapeutic hypothermia (TH) used and risk factors associated with mortality in these high-risk neonates.

## Materials and Methods

### Study Design

This was an observational retrospective cohort study. The inclusion criteria were all LPTN diagnosed to have HIE and admitted to the NICUs of MNNR between 1 January 2016 and 31 December 2019. The exclusion criteria were gestation of <35 weeks, death in delivery rooms, major malformations, congenital infections, or inborn errors of metabolism. The following data of each recruited neonate was extracted from the MNNR database: demographic characteristics (birth weight, gestation, gender, ethnic group, inborn/outborn, singleton/multiple, mode of delivery), Apgar score at 5 min, admission temperature, highest Thompson score before 6 h of life, HIE severity (mild, moderate, and severe), cooling therapy given (yes, no), type of TH [cooling blanket/cap, passive cooling ± gel packs (P±CGP), both (defined as initially cooled by P±CGP until a set of SCD was available for TH)], late-onset sepsis (LOS) (yes, no), pneumothorax (yes, no), and outcome at discharge (alive or dead). For centers without rectal thermistor probes for monitoring rectal temperature continuously, digital thermometers were used for intermittent measurement of axilla temperature of neonates undergoing TH using P±CGP. During this study, we also conducted a short questionnaire survey of all NICUs on the types and number of sets of TH devices, and methods for monitoring core temperature used during this 4-year period.

### Definitions

The following were definitions published annually in a manual provided by the MNNR guiding health care providers in diagnosing and submitting data to the MNNR. Gestation was reported in complete weeks based on antenatal ultrasound, maternal last menstrual period, or the new Ballard score after birth (15). HIE was diagnosed in any neonates with all of the following three criteria: (a) any of three clinical features of encephalopathy within 72 h after birth (abnormal level of consciousness, abnormal muscle tone, abnormal deep tendon reflexes, seizures, abnormal Moro reflex, abnormal sucking reflex, abnormal respiratory pattern, and oculomotor or pupillary abnormalities); (b) three or more findings of acute perinatal events (evidence of fetal distress on antepartum monitoring, cord arterial pH < 7.00, 5-min Apgar score <5, evidence of multi-organ system dysfunction within 72 h of life, abnormal electroencephalogram, and abnormal brain imaging showing ischemia/edema; and (c) absence of underlying congenital cerebral abnormalities/infections or inborn errors of metabolism. The severity of HIE was classified as mild, moderate, or severe according to Sarnat’s criteria (16) shortly after birth before 6 h of life. Neonates, who were alert or hyperalert with a normal or exaggerated response to arousal, and no seizures were classified as mild HIE; those with lethargy and decreased response to arousal, with or without a seizure, as moderate HIE; and those in deep stupor or coma at birth and not arousable as severe HIE. LOS was diagnosed in neonates developing symptoms of sepsis after 72 h of life associated with a positive blood culture. The annual incidence of HIE in the MNNR was calculated by dividing the total number of inborn HIE neonates by the total live births in all participating hospitals of the respective years. For this study, NICUs were categorized as small (<50), medium-sized (50–100), and large (>100), based on the number of HIE LPTNs admitted during these 4 years.

### Ethics Approval

Parental consent was not obtained as all data were anonymized. Ethical clearance for this study was granted by the Malaysian Ministry of Health and registered under the National Medical Research Registry (NMRR-05-04-168).

### Statistical Analysis

Categorical variables were reported as number and percentage, and continuous variables as mean (±SD) or median (range) where appropriate. Demographic, clinical, and outcome data of neonates receiving TH using SCD, P±CGP, or no TH were compared in each category of the severity of HIE. For between-group analysis, the Chi-square test was used for categorical variables, and Student *t*-test for continuous variables, and the Kruskal–Wallis test for among group analysis. Simple and multiple logistic regression analyses were performed to identify significant risk factors associated with in-hospital mortality. The potential risk factors examined were birth weight, gestation, gender, ethnic groups, modes of delivery, inborn/outborn, 5-min Apgar score <5, admission temperature, LOS, pneumothorax, number of HIE neonates admitted per center, types of TH received, the severity of HIE, and Thompson scores. Multicollinearity among independent variables was checked by calculating the variance inflation factor (VIF); variables with a VIF of >3 were removed from the final model. *P*-values of <0.05 were considered statistically significant; all tests were two-sided. Statistical package SPSS v.27 was used for analysis.

## Results

### Demographic Data

**FIGURE 1:**
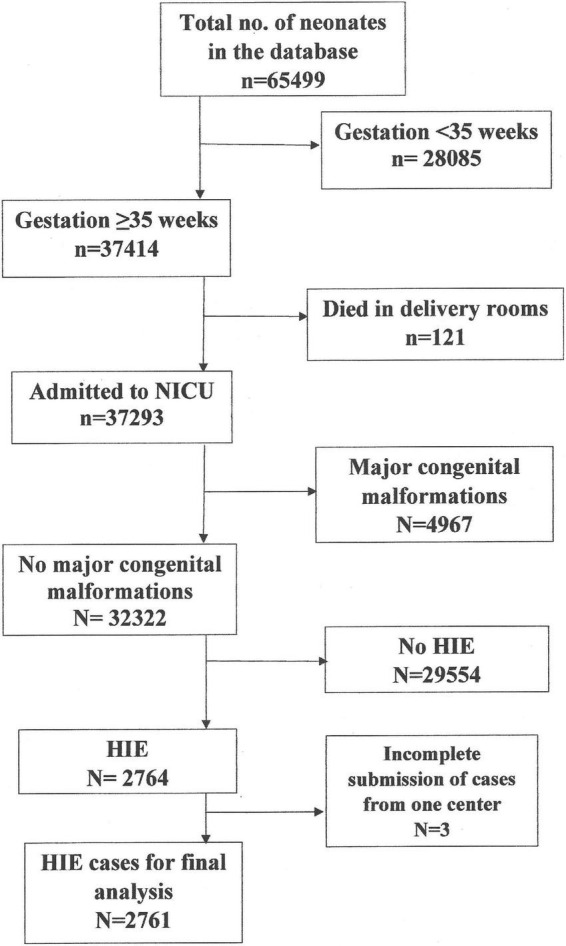
Recruitment flowchart.

There were 2,761 LPTN with HIE admitted to the 44 NICUs (Figure 1); 34.8% of LPTN had mild, 44.6% had moderate, and 20.6% had severe HIE. The majority of neonates in this cohort were Malays, singletons, and inborn (Table 1). The annual incidence of inborn HIE (per 1,000 live births) was 2.2 in 2016, 2.3 in 2017, 2.4 in 2018, and 2.32 in 2019. When compared with inborn HIE neonates, outborns had a lower proportion of mild HIE (20.6% vs. 36.7%), a similar proportion of moderate HIE (46% vs. 44.4%), but a higher proportion of severe HIE (33.4% vs. 18.9%) (*p* < 0.001). A significantly lower proportion of outborns received TH than the inborn (61.3% vs. 66.9%, *p* = 0.045).

**TABLE 1:** Demography and outcome of hypoxic-ischemic encephalopathy (HIE) neonates in the Malaysian National Neonatal Registry (MNNR), 2016–2019.

Variables	HIE stages based on Sarnat criteria			
	Total *N* = 2,761	Mild *N* = 960	Moderate *N* = 1,232	Severe *N* = 569
Thompson score median (IQR)	*N* = 2,755 8 (5,12)	*N* = 958 5 (2, 7)	*N* = 1,229 9 (7, 11)	*N* = 568 13 (10,16)
Gestation, mean (95% CI) weeks	38.6(38.5,38.6)	38.6(38.5,38.7)	38.7(38.6,38.7)	38.5(38.5,38.6)
Birth weight, mean (95% CI) g	3,019(3,000,3,037)	2,994(2,863,3,025)	3,050(3,024,3,076)	2,992(2,948,3,035)
Males, *n* (%)	1718 (62.2)	609 (63.4)	761 (61.8)	348 (61.2)
Ethnic groups, *n* (%)				
Malay	1791 (64.9)	626 (65.2)	876 (71.1)	289 (50.8)
Chinese	152 (5.5)	60 (6.3)	58 (4.7)	34 (6.0)
Indian	105 (3.8)	46 (4.8)	43 (3.5)	16 (2.8)
Orang Asli	25 (0.9)	7 (0.7)	12 (1.0)	6 (1.1)
Sabah native	211 (7.6)	69 (7.2)	70 (5.7)	72 (12.7)
Sarawak native	90 (3.3)	33 (3.4)	35 (2.8)	22 (3.9)
Other Malaysian	19 (0.7)	5 (0.5)	6 (0.5)	8 (1.4)
Foreigner	368 (13.3)	114 (11.9)	132 (10.7)	122 (21.4)
Singleton, *n* (%)	2707 (98.1)	940 (98.0)	1212 (98.4)	555 (97.5)
Inborn, *n* (%)	2435 (88.2)	893 (93.0)	1082 (87.8)	460 (80.8)
In-hospital mortality, *n* (%)	338 (12.2)	18 (1.9)	51 (4.1)	269 (47.3)
Median duration of hospitalization of all neonates, in days (IQR)	8(5,13)	6(5,10)	9(6,14)	9(3,19)

*CI, confidence intervals; HIE, hypoxic-ischemic encephalopathy; IQR, interquartile range.*

### Types of Cooling Devices and Therapeutic Hypothermia Rates

All NICUs provided some form of TH to their HIE neonates. Twenty-four (54.5%) NICUs had SCD (Table 2). Most (4/5, 80%) large NICUs and smaller NICUs (43.5%) had SCD. Of the total 1,830 (66.3%) neonates receiving TH, 38.5% were by SCD. The most common type of SCD used was cooling blankets (TECOtherm Neo, Tec Com Medizintechnik GMBh, Kabelsketal, Germany, or Artic Sun 5000 Temperature Management System, C.R.Bard, Inc., Louisville, CO, United States). Only one NICU had a set of Cooling Cap (Olympic Cool-Cap cooling system, Natus Medical, Inc., Seattle, WA, United States). The majority of the neonates treated with SCD were admitted to large NICUs. All NICUs monitored rectal temperature during passive TH except in six non-SCD centers.

**TABLE 2:** Frequency distribution of cooling devices and number of neonates with hypoxic ischemic encephalopathy in different types of neonatal intensive care units (NICUs), 2016–2019.

Variables	Small NICU	Medium sized NICU	Large NICU	Overall
No. of NICUs	23	16	5	44
Total no. of HIE neonates admitted (%) during the 4 years	689(25.0)	1,057(38.3)	1,015(36.8)	2,761 (100)
No. of HIE neonates admitted per NICU, median (range) during the 4 years	36(1−49)	64.5(51−95)	128(106−436)	49 (1-436)
No. of NICU with different types of TH devices (%)				
Had SCD only	8 (34.8)	6 (37.5)	2 (40)	16 (36.4)
Had gel packs only	8 (34.8)	3 (18.8)	1 (20)	12 (27.3)
Had both SCD and gel packs	2 (8.7)	4 (25.0)	2 (40)	8 (18.2)
Had no devices	5 (21.7)	3 (18.8)	0 (0)	8 (18.2)
No. of NICUs with SCD per NICUs (%)				
0 set	13 (56.5)	6 (37.5)	1 (20)	20 (45.5)
1 set	8 (34.8)	7 (43.8)	0	15 (34.1)
2 sets	2 (8.7)	3 (18.8)	4 (80)	9 (20.5)
No. of neonates treated with				
SCD only (%)	115 (16.7)	335 (31.7)	613 (60.4)	1063 (38.5)
P±CGP only (%)	290 (42.1)	300 (28.4)	80 (7.9)	670 (24.3)
Both methods (%)	44 (6.4)	43 (4.1)	10 (1.0)	97 (3.5)
No treatment (%)	240 (34.8)	379 (35.9)	312 (30.7)	931 (33.7)

*NICU, neonatal intensive care units; HIE, hypoxic-ischemic encephalopathy; TH, therapeutic hypothermia; SCD, servo-controlled device.*

**FIGURE 2:**
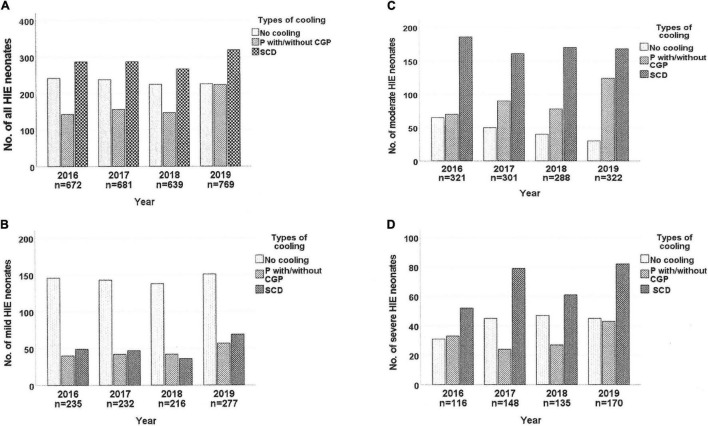
Trends of therapeutic hypothermia used for neonates with hypoxic-ischemic encephalopathy in the MNNR between 2016 and 2019. HIE, hypoxic-ischemic encephalopathy; P with/without CGP, passive cooling with/without uses of cooled gel packs; SCD, servo-controlled devices.

Figure 2 shows that more HIE neonates were admitted in 2019 than in previous years. The annual TH rates remained static until 2019 when significantly more were cooled using P±CGP (2016: 24.1%, 2017: 26.7%, 2018: 26.9%, 2019: 32.6%; *p* = 0.024), irrespective of HIE severity. There was no significant increase in use of SCD (2016: 39.9%, 2017: 38.3%. 2018: 37.9%, 2019: 38%).

Table 3 shows that the median Thompson scores were higher in the more severe grades of HIE; 39.8% (382/960) of mild, 85% (1,047/1,232) of moderate, and 70.5% (401/569) of severe HIE neonates received TH; and only 51.6% of moderate and 43.6% of severe HIE received TH using SCD.

**TABLE 3:** Comparison of demographic, clinical and outcome characteristics of neonates with mild, moderate, and severe HIE (based on Sarnat Criteria) treated with different types of therapeutic hypothermia.

Variables	Mild HIE *N* = 960			Moderate HIE *N* = 1232			Severe HIE *N* = 569		
	No TH *N* = 578	*^a^*P±CGP *N* = 203	SCD *N* = 179	No TH *N* = 185	*^b^*P±CGP 411	SCD *N* = 636	No TH *N* = 168	*^c^*P±CGP *N* = 153	SCD *N* = 248
Thompson scores median (IQR)	*N* = 577 4 (2, 6)	6(4,8)	*N* = 178 7 (5, 9)^†^	9(7,11)	9(7,11)	*N* = 6339(7,12)^§§^	14(10,16)	*N* = 152 13 (10, 17)	13(9−16)
Birth weight, g, mean (SD)	2,994(494)	2,996(481)	2,992 (494)	3,041(472)	3,017(447)	3,074(487)	2,923(512)	2,984(507)	3,042(542)
Gestation, weeks, median (IQR)	39(38,40)	39(37,40)	39 (38, 39)	39(38,40)	39(38,40)	39(38,40)	39(37,40)	39(38,40)	39(38,40)
Males, *n* (%)	363 (62.8)	126 (62.1)	120 (67.0)	113 (61.1)	230 (56.0)	418 (65.7)	88 (52.4)	98 (64.1)	162 (65.3)
Ethnic groups, *n* (%)									
Malay Malaysian	367 (63.5)	142 (70.0)	117 (65.4)	122 (65.9)	283 (68.9)	471 (74.1)	98 (58.3)	82 (53.6)	109 (44.0)
Other Malaysian	138 (23.9)	42 (20.7)	40 (22.3)	42 (22.7)	79 (19.2)	103 (16.2)	42 (25.0)	37 (24.2)	79 (31.9)
Foreigner	73 (12.6)	19 (9.4)	22 (12.3)	21 (11.4)	49 (11.9)	62 (9.7)	28 (16.7)	34 (22.2)	60(24.2)**
LSCS, *n* (%)	194 (33.6)	68 (33.5)	49 (27.4)	73 (39.5)	123 (29.9)	160(25.2)^§^	82 (48.8)	71 (46.4)	111 (44.8)
Outborns, *n* (%)	45 (7.8)	14 (6.9)	8 (4.5)	38 (20.5)	33 (8.0)	79(12.4)^§^	43 (25.6)	25 (16.3)	41(16.5)**
5-min Apgar score, median (IQR)	6(5,8)	6(4,7)	6 (5, 7)^†^	*N* = 182 6 (4,7.3)	*N* = 402 5 (4, 6)	*N* = 6295(4,6)^§^	*N* = 152 3 (1, 5)	*N* = 148 4 (2,5)	*N* = 227 4 (2, 5)
Type of NICU, *n* (%)									
Small	164 (28.4)	102 (50.2)	32 (17.9)^†^	36 (19.5)	162 (39.4)	53 (8.3)	40 (23.8)	70 (45.8)	30 (12.1)
Medium size	255 (44.1)	79 (38.9)	56 (31.3)	67 (36.2)	205 (49.9)	186 (29.2)	57 (33.9)	59 (38.6)	93 (37.5)
Large	159 (27.5)	22 (10.8)	91 (50.8)	82 (44.3)	44 (10.7)	397(62.4)^§^	71 (42.3)	24 (15.7)	125(50.4)*
Admission temperature, median (IQR) °C	36.4(35.8,36.7)	36.1(35.5,36.6)	36.0	36.0(35.1,36.5)	36.0(34.6,36.5)	35.6(34.5,36.4)§	35.5(34.2,36.3)	35.4(34.0,36.4)	35.3(34.3,36.1)
			(35.0, 36.5)^†^						
LOS, *n* (%)	4 (0.7)	0	2 (1.1)	2 (1.1)	3 (0.7)	6 (0.9)	5 (3.0)	3 (2.0)	3 (1.2)
Pneumothorax, *n* (%)	29 (5.0)	7 (3.4)	11 (6.1)	15 (8.1)	21 (5.1)	23(3.6)^§§^	13 (7.7)	13 (8.5)	15 (6.0)
Died, *n* (%)	12 (2.1)	3 (1.5)	3 (1.7)	22 (11.9)	16 (3.9)	13(2.0)^§^	117 (69.6)	74 (48.4)	78(31.5)*
Age of death, median (IQR) days	15(1.0,13.3)	1(1,1)	2 (1, 2)	2(1,5.3)	2(1,2)	5(2.5,19.0)^§§^	1(1,4)	3(2,8)	4(2,7.3)*
Duration of hospital stay of survivors, median (IQR) days	6(4,9)	7(6,11)	8 (6, 11)^†^	11(8,17)	10(7,14)	9(6,14)^§^	23(12,36)	21(13,28)	15(9,22)*

HIE, hypoxic-ischemic encephalopathy; TH, therapeutic hypothermia; SCD, servo-control device; P±CGP, passive cooling with/without cool gel pack; LSCS, lower segment Cesarean section; LOS, late-onset sepsis; SD, standard deviation; IQR, interquartile range. The P±CGP groups in this table also included those neonates cooled with both methods: ^a^n = 22; ^b^n = 46; ^c^n = 44. Comparison of variables among neonates receiving different types of TH in each category of HIE: Severe HIE, *p < 0.001, **p < 0.05; Moderate HIE, ^§^p < 0.001, ^§§^p < 0.05; Mild HIE: ^†^P < 0.001.

### In-Hospital Mortality

A total of 338 (12.2%) deaths was reported, comprising 1.9% (18/960) of the mild, 4.1% (51/1,232) of the moderate, and 47.3% (269/569) of the severe HIE neonates (Table 3). In mild HIE neonates, there was no significant difference in mortality rates irrespective of the types of TH used (no TH: 2.1%, P±CGP: 1.5%, SCD: 1.7%; *p* = 0.696). In moderate HIE neonates, mortality rates were significantly lower with SCD and P±CGP than “no TH”(*p* < 0.001). In severe HIE neonates, mortality was significantly lower in those given SCD than with P±CGP (*p* = 0.002) and “no TH” (*p* < 0.001). The most common cause of death reported in each grade of HIE was HIE and not all of them had Thompson score ≥7 (Table 4).

**TABLE 4:** Causes of death of hypoxic-ischemic encephalopathy according to the severity.

Causes of death	Mild HIE *N* = 18 (%)		Moderate HIE *N* = 51 (%)		Severe HIE *N* = 269 (%)		All HIE *N* = 338 (%)
	Death	Thompson score ≥7	Death	Thompson score ≥7	Death	Thompson score ≥7	
HIE	8 (44.4)	4	27 (52.9)	23	251 (93.3)	241	286 (84.6)
MAS	3 (16.7)	1	7 (13.7)	6	0	0	10 (3.0)
PPHN	0	0	2 (3.9)	2	0	0	2 (0.6)
Sepsis	0	0	6 (11.7)	5	3 (1.1)	3	9 (2.7)
Pneumonia	1 (5.6)	1	3 (5.9)	3	1 (0.4)	1	5 (1.5)
Pneumothorax	0	0	1 (2.0)	1	0	0	1 (0.3)
NEC perforated	1 (5.6)	0	0	0	0	0	1 (0.3)
IVH	1 (5.6)	0	0	0	0	0	1 (0.3)
Twin-twin transfusion	0	0	1 (2.0)	0	0	0	1 (0.3)
Myopathy	0	0	1 (2.0)	1	0	0	1 (0.3)
Not stated	4 (22.2)	3	3 (5.9)	2	14 (5.2)	12	21 (6.2)

*HIE, hypoxic-ischemic encephalopathy; MAS, meconium aspiration syndrome; PPHN, persistent pulmonary hypertension; NEC, necrotizing enterocolitis; IVH, intraventricular hemorrhage.*

Multiple logistic regression analysis shows after controlling for various potential confounders listed in Table 5, 5-min Apgar score <5, Cesarean delivery, foreigners, pneumothorax, TH in centers admitted <100 HIE neonates, TH by P±CGP, receiving no TH, moderate or severe HIE, and Thompson scores ≥7 were the significant risk factors associated with mortality. The regression model has a Nagelkerke R square of 0.503, Hosmer and Lemeshow test *p* = 0.425, and Omnibus test of model *p* < 0.001. The area under the receiver operating characteristics curve (ROC) for the predictive model is 0.912 (95% CI: 0.894, 0.930), *p* < 0.0001.

**TABLE 5:** Simple and multiple logistic regression analyses of potential risk factors associated with in-hospital mortality in all HIE neonates in the Malaysian National Neonatal Registry (MNNR), 2016–2019.

Variables	In-hospital mortality *N* = 338	Alive *N* = 2423	Odds Ratio (95% CI)	Adjusted Odds Ratio (95% CI)
Birth weight, mean (SD) g	2,953 (533)	3,027 (484)	1.000 (0.999, 1.000)	1.000 (1.000,1.000)
Gestation, mean (SD) weeks	38.4 (1.6)	38.6 (1.4)	0.883 (0.816, 0.955)	0.969 (0.862, 1.089)
**Gender**				
Females	141	902	1	1
Males	197	1521	0.829 (0.657, 1.044)	1.043 (0.759, 1.433)
**Ethnic groups**				
Non-Malay Malaysians	71	531	1	1
Malay Malaysians	194	1597	0.909 (0.680, 1.213)	1.222 (0.831, 1.798)
Foreigners	73	295	1.851 (1.296, 2.643)	1.646 (1.006, 2.692)
**Modes of delivery**				
Vaginal route	151	1679	1	1
Cesarean section	187	744	2.795 (2.218, 3.522)	2.335 (1.700, 3.207)
**Birthplace**				
Inborn	276	2159	1	1
Outborn	62	264	1.837 (1.356, 2.489)	1.063 (0.665, 1.700)
**5-min Apgar score**				
5–10	102	1610	1	1
<5	213	757	4.441 (3.454, 5.710)	1.436 (1.019, 2.023)
Missing data	23	56	–	
**Admission temperature**, *n*				
>36.6	51	564	1	1
35.0–36.5	160	1314	1.347 (0.968, 1.874)	0.899 (0.579, 1.394)
33.0-<35.0	101	491	2.275 (1.591, 3.253)	1.184 (0.722, 1.944)
<33.0	26	54	5.325 (3.076, 9.216)	1.1143 (0.538, 2.427)
**Late-onset sepsis**				
No	328	2405	1	1
Yes	10	18	4.074 (1.864, 8.900)	2.889 (0.879, 9.496)
**Pneumothorax**				
No	296	2318	1	1
Yes	42	105	3.132 (2.147, 4.570)	3.435 (1.996, 5.914)
**No. of HIE cases admitted**				
≥100	98	917	1	1
50-<100	142	915	1.452 (1.105, 1.908)	1.552 (1.065, 2.260)
<50	98	591	1.552 (1.151, 2.091)	1.898 (1.225, 2.940)
**Type of Therapeutic hypothermia**				
Servo-control devices	109	1051	1	1
Passive cooling ± gel packs	78	592	1.270 (0.934, 1.728)	1.553 (1.031, 2.338)
No TH	151	780	1.867 (1.435, 2.429)	4.749 (3.201, 7.045)
**Severity of HIE, *n***				
Mild	18	942	1	1
Moderate	51	1181	2.260 (1.312, 3.894)	2.823 (1.495, 5.333)
Severe	269	300	46.926 (28.62, 76.95)	34.925 (18.478, 66.012)
**Thompson scores**				
<7	29	844	1	1
7–13	132	1309	2.935 (1.945, 4.427)	1.776 (1.023, 3.082)
≥14	176	265	19.329 (12.747, 29.311)	3.641 (2.000, 6.629)
Missing data	1	5	–	

*HIE, hypoxic-ischemic encephalopathy; SD, standard deviation; CI, confidence intervals; TH, therapeutic hypothermia.*

## Discussion

In this observational retrospective cohort study of HIE neonates in the MNNR, we found that all Malaysian NICUs provided some form of TH using either SCD or P±CGP, as only 55.4% of our NICUs had SCD. TH using SCD was received by 18.6% of mild, 51.6% of moderate, and 43.6% of severe HIE neonates. Multiple logistic regression analysis identified several factors to be significantly associated with mortality, including the severity of HIE, no TH, and TH using P±CGP.

Presently, the most reliable methods to diagnose and grade HIE are magnetic resonance imaging (MRI) and electroencephalogram (EEG). However, the majority of the Malaysian NICUs did not have timely access to MRI services during the study period, and only 22/44 NICUs had amplitude-integrated EEG (aEEG). The Sarnat score and Thompson scores were (and still are) used for diagnosis and management of HIE by the managing neonatologists in our NICUs. The findings in the present study suggested that both the Sarnat score and Thompson score could have misclassified some of the neonates, as we found some mild HIE neonates had high Thompson scores of ≥7 and died due to HIE (Table 4); and some moderate and severe HIE neonates who died due to HIE had low Thompson scores.

Many of the HIE neonates were treated with P±CGP in our NICUs. Although this resourceful method was commendable, multiple logistic regression analysis in our study showed that, after controlling for various potential confounders, its use was associated with a significantly higher (>1.5 times) risk of mortality than SCD. The major problems of using P±CGP include difficulty in maintaining a constant core temperature and labor-intensiveness. A neonate on P±CGP needs a longer time to achieve the target core temperature (about 1 h). Each gel packs in-use needs to be changed every 3–4 h to maintain the target temperature. Some NICUs used axillary temperature as a surrogate for monitoring core temperature which could further compromise the effectiveness of P±CGP, as the axillary temperature does not reflect core temperature accurately.

According to current management guidelines (8, 13), TH was indicated only for neonates with moderate and severe HIE. In this cohort, there were 18.6% of mild HIE neonates treated with TH using SCD. The benefits of TH in mild HIE are still controversial and side effects have been reported (17, 18). Yet, at the same time, there were 353 (19.6%) moderate or severe HIE neonates receiving no TH. It is uncertain whether this was due to their failure to meet the criteria for TH, or the ignorance of some staff on the benefits of TH. The significant increase in TH during the fourth year (2019) suggested that perhaps more NICU staff were aware of these benefits recently. However, because of the inadequate number of SCD, this increasing trend of TH was reflected mainly by increased use of P±CGP.

During the study period, there were only five large NICUs; they had more sets of SCD per NICU than many smaller NICUs and treated more neonates with SCD and had lower mortality rates. Their better outcome could be attributed to be having overall better equipment and expertise than many smaller NICUs. These findings suggest that the regionalization of TH in Malaysia may lead to better outcomes for HIE neonates in this country. However, for TH to be effective, it must be administered within the first 6 h of life (5, 6, 8). Like many LMICs, traffic jams and/or inadequate neonatal transportation services in Malaysia are common barriers to ensure high-risk outborns reaching regional NICUs for TH within 6 h of birth. A second option to improve outcomes is to equip all Malaysian NICUs with an adequate number of sets of SCD to provide effective TH equitably and timely to any neonate who needs it.

Currently, all NICUs provided care to all grades of HIE. Our findings suggest that to improve outcomes of HIE neonates in Malaysia, equipping NICUs with adequate sets of SCD, supportive equipment, and trained personnel, and improving access to diagnostic facilities for HIE should be considered.

Our real-world findings concur with those reported from HICs that TH was associated with better survival in both moderate and severe HIE neonates (8). Our results contrasted with those of a multicenter randomized controlled study (the HELIX study) carried out in several NICUs in three LMICs, which reported significantly higher mortality associated with TH than no TH (19). Besides the difference in the design of their study, other possible reasons for the differences in the outcome of TH between the present study and the HELIX study include the relatively smaller sample size, different diagnostic criteria used, and a much higher proportion (80%) of outborn neonates recruited in the HELIX study.

Many studies from HICs reported better long-term neurodevelopmental outcomes and cost-effectiveness in survivors of moderate HIE following TH (20, 21). In Malaysia, there is a need to carry out similar studies to confirm the benefits of TH on these high-risk neonates, including comparing the outcome of HIE neonates treated with P±CGP vs. SCD, like studies reported in other LMICs (11, 22).

The strengths of this study include its large sample size, inclusion of all HIE neonates from all the major NICUs in a middle-income country, systematic and prospective collection of patients using a standard format, and comparison of mortality of HIE receiving different types of TH in a real-world situation. The major limitations of this study are not a randomized controlled study; no information included in the MNNR database on each neonate’s Apgar score at 10 min, mechanical ventilation or resuscitation at 10 min, cord pH, and clinical features of HIE for us to verify the diagnostic accuracy of HIE in this study; no information in the database on the timing of Sarnat examination, seizures prevalence during the first 6 h of life, actual age, and indications for commencement of TH, age of initiation and duration of TH, adverse effects of TH, brain MRI findings, and neurological findings at discharge.

## Conclusion

Both SCD and P±CGP were used for TH in Malaysian NICUs. Moderate/severe HIE, TH using P±CGP, and receiving no TH were among the risk factors associated with mortality.

## Data Availability Statement

The original contributions presented in this study are included in the article/supplementary material, further inquiries can be directed to the corresponding author/s.

## Ethics Statement

The studies involving human participants were reviewed and approved by the National Medical Research Registry of the Ministry of Health of Malaysia. Written informed consent from the participants’ legal guardian/next of kin was not required to participate in this study in accordance with the National Legislation and the Institutional Requirements.

## Author Contributions

NB: conceptualization, data analysis, and manuscript preparation. NB, SN, and SC: data extraction and review and editing of the manuscript. All authors contributed to the article and approved the submitted version.

## Conflict of Interest

The authors declare that the research was conducted in the absence of any commercial or financial relationships that could be construed as a potential conflict of interest.

## Publisher’s Note

All claims expressed in this article are solely those of the authors and do not necessarily represent those of their affiliated organizations, or those of the publisher, the editors and the reviewers. Any product that may be evaluated in this article, or claim that may be made by its manufacturer, is not guaranteed or endorsed by the publisher.
